# Reduction in Door-to-Groin Puncture Time for Endovascular Treatment in Acute Ischemic Stroke Patients With Large Vessel Occlusion

**DOI:** 10.7759/cureus.28348

**Published:** 2022-08-24

**Authors:** Mudassir Farooqui, Sajid Suriya, Syed Quadri, Aqsa Baig, Mohammad Hamza Khalil, Ayesha Liaquat, Asif Taqi

**Affiliations:** 1 Neurology, University of Iowa Hospitals and Clinics, Iowa City, USA; 2 Neurology, University of New Mexico School of Medicine, Albuquerque, USA; 3 Neurology, Massachusetts General Hospital, Harvard Medical School, Boston, USA; 4 Neurology, Liaquat National Hospital and Medical College, Karachi, PAK; 5 Neurology, Beth Israel Deaconess Medical Center, Boston, USA; 6 Medicine and Surgery, Karachi Medical and Dental College, Karachi, PAK; 7 Neurological Surgery, Vascular Neurology of Southern California, Thousand Oaks, USA

**Keywords:** large vessel occlusion, stroke scale, thrombectomy, door to groin puncture time, ischemic stroke

## Abstract

Background

The outcome of mechanical thrombectomy for large vessel occlusion (LVO) in patients with acute ischemic stroke (AIS) is time-dependent. In the current stroke workflow, the pre-hospital delay is one of the most common reasons for an increase in door-to-groin puncture time (DGPT). In the present study, we sought to compare the difference in (DGPT) before and after the implementation of the Ventura Emergent Large Vessel Occlusion Score (VES) protocol for LVO.

Methods

VES was implemented in the Ventura County of California by Emergency Medical Services (EMS). We performed a retrospective analysis to compare DGPT of patients undergoing endovascular treatment (EVT) pre- and post-VES implementation. Mean and standard deviation was reported for the continuous variable ‘time for intra-arterial (IA) treatment’ in minutes. The Mann-Whitney test was used for the comparison of the variable between the two groups. analyses were performed using SAS v9.4 (SAS Institute Inc., Cary, NC) with a significant p-value of ≤0.05.

Results

A total of 304 (males: 142 and females: 162) patients were alerted of the stroke code by the EMS. VES was positive in 139 patients. Of these, 64 (46%) were males and 75 (54%) were females. VES score of 1, 2, 3, and 4 were recorded in 57 (41%), 44 (31.6%), 31 (22.3%), and 7 (5%) patients, respectively. A total of 48 VES-positive patients underwent EVT. There were 62 patients who underwent EVT before the implementation of the VES protocol. The mean DGPT for the EVT among post-VES patients was 65 minutes, which was significantly (p=0.0009) shorter than the mean DGPT of 109 minutes among pre-VES patients.

Conclusion

VES is a simplified and effective tool for identifying LVO in the field. Implementation of VES showed significantly reduced DGPT in LVO patients.

## Introduction

Acute ischemic stroke (AIS) is one of the leading causes of death and long-term disability [1,2]. Large vessel occlusion (LVO) strokes represent 30-40% of all cases, resulting in approximately 90% of stroke-related morbidity and mortality [2]. Stroke treatment is time sensitive and depends on swift reperfusion of the ischemic brain tissue. Treatment of AIS-LVO involves both intravenous tissue plasminogen activator (IV-tPA) and endovascular treatment (EVT). Both treatments decrease functional disability when given early after symptom onset, however, their effectiveness drops dramatically with time [1,3,4]. Where IV-tPA is effective in the first 4.5 hours, mechanical thrombectomy (MT) is proven to be beneficial up to 24 hours from the symptom onset [3,4]. 

Stroke care is facilitated through coordinated systems encompassing prehospital identification and a regionalized network of hospitals to provide rapid diagnosis, triage, and treatment [5]. In the current stroke model, patients are directed to the nearest community centers, evaluated, and then transferred to an EVT-capable center for suspected or confirmed LVO [5,6]. Recently, there has been a focus on early identification and reducing stroke time metrics to facilitate timely treatment. Of these, door-to-groin puncture time (DGPT) has a significant impact on the functional outcomes [7]. DGPT for interhospital transfer patients is significantly shorter than for patients who arrive by direct transfer through emergency medical services (EMS) because of early identification and notification from the transferring facilities [8]. The major impediment at the pre-hospital level is due to delayed identification of LVO, advanced notifications, and transportation to the Comprehensive Stroke Center (CSC) [9,10].

Several pre-hospital stroke scales have been developed to identify LVO-AIS in the field by the emergency medical services (EMS) [11-16]. While these scales have variable sensitivity and specificity, they can decrease uncertainty in risk-stratifying patients for LVO and augment triage decision-making. Recently a simplified tool, Ventura Emergent Large Vessel Occlusion Score (VES) was introduced to identify LVO in the field. VES is being used by EMS of Ventura County in California, USA, to assess stroke patients for LVO as part of pre-hospital assessment [17]. In the current study, we reviewed the impact of the VES scale and LVO triage in the field on the DGPT. The purpose of our study is to compare the difference in DGPT before and after the implementation of the VES protocol. The study data was presented and published at the American Academy of Neurology (AAN) annual meeting 2021.

## Materials and methods

Study design

VES was implemented in the Ventura County of California in January 2016, as an attempt to improve the triage process and reduce the pre-hospital delay for possible LVO patients. The current study is a retrospective analysis of a prospectively collected database. The current retrospective study was exempted from full board review by the Western Institutional Review Board (WIRB) under 45 CFR 46.101(b) (4) and is reported in accordance with the Strengthening the Reporting of Observational Studies in Epidemiology (STROBE) guidelines [18].

Ventura emergent large vessel occlusion (ELVO) score

Ventura ELVO Score (VES) consists of aphasia, neglect, eye deviation, and obtundation (Table 1). The total score ranges from 0-4 for each of the component’s categories (yes or no). VES was considered positive (score of 1) if either of the components was positive. VES was used in conjunction with Cincinnati pre-hospital stroke scale (CPSS) by an EMS personnel [17]. Patients were screened with CPSS and VES in the field and were considered LVO stroke codes when VES was positive, which then activated the neurointerventional team at the receiving hospital for possible EVT.

**Table 1:** Ventura ELVO score ELVO: Emergent Large Vessel Occlusion

Assessment parameter	Characteristics	Score
Eye deviation	Forced deviation of both eyes to either side	Positive = 1 Negative = 0
Aphasia	The patient is awake but one or more of the following is present: 1) Unable to repeat a sentence, 2) Unable to name an object, 3) Talking gibberish and/or not following any commands, 4) Mute	Positive = 1 Negative = 0
Neglect	Identified by individual then simultaneous stimulus. (If the patient can feel both sides individually but not feeling one side on simultaneous stimulation then it's positive)	Positive = 1 Negative = 0
Obtundation	Positive if the patient is not staying awake during the conversation	Positive = 1 Negative = 0

Patient population

Data were collected using electronic medical records of Los Robles Hospital and Medical Center (LRHMC) and EMS run sheets of Ventura County Emergency Medical Services (VCEMS). All the stroke code patients ≥18 years were eligible. This includes all the patients brought in by VCEMS and underwent EVT before and after implementation of the VES protocol. Patients who were VES positive in the field were confirmed for LVO before proceeding to EVT. All the patients were validated by using neuroimaging.

Statistical analysis

All the data were collected using Microsoft Excel. Mean and standard deviation were reported for the continuous variable ‘time for intra-arterial (IA) treatment’ in minutes. Normality was assessed using the Shapiro-Wilk’s test which indicated that the variable is not normally distributed, therefore, the Mann-Whitney U-test was used for the comparison of the variable between the two groups. A P-value of ≤0.05 was considered statistically significant for the test. Analysis was performed using SAS v9.4 (SAS Institute Inc., Cary, NC). The data will be made available from the corresponding author on reasonable request.

## Results

A total of 304 patients were alerted of the code stroke by the EMS during the period January 01, 2016, to March 31, 2018, after the implementation of VES protocol. Of these, there were 142 (47%) males and 162 (53%) females. VES was positive in 139 patients. Out of those who were positive for VES, there were 64 (46%) males and 75 (54%) females. Among these, the VES of 1, 2, 3, and 4 were recorded in 57 (41%), 44 (31.6%), 31 (22.3%), and 7 (5%) patients respectively (Table 2).

**Table 2:** Distribution of scores among VES-positive stroke code patients. VES: Ventura Emergent Large Vessel Occlusion (ELVO) Score

VES (N=139)	No of the patients (%)
Male	64 (46)
Females	75 (54)
1	57 (41)
2	44 (31.6)
3	31 (22.3)
4	7 (5)

**Figure 1:**
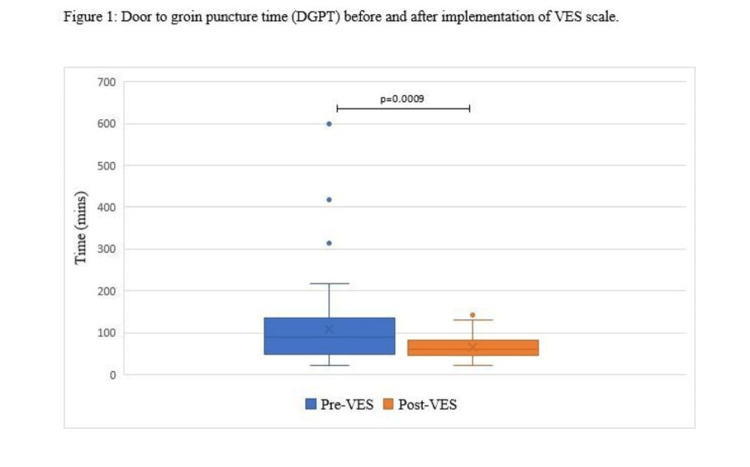
Door-to-groin puncture time (DGPT) before and after implementation of VES scale. Box and whisker plot represent the door to groin puncture times for the patients before and after implementation of the VES scale. Mean, median and interquartile (IQR) ranges are represented. Blue and orange dots above the IQR are outliers. VES: Ventura Emergent Large Vessel Occlusion (ELVO) Score

A total of 48 patients who were positive for VES underwent intra-arterial thrombectomy. There were 62 patients who underwent intra-arterial thrombectomy before the implementation of VES protocol and which were spanned over the period of September 2014 to December 2015. The mean DGPT for the IA treatment among post-VES patients was 65 minutes, which was significantly shorter (p=0.0009) as compared to the mean DGPT of 109 minutes among pre-VES patients (Figure 1).

## Discussion

Early reperfusion therapy with thrombolysis or mechanical thrombectomy has shown better functional outcomes with decreased morbidity and disability [3,19]. Studies have also observed that pre-hospital delay is a major contributor to initiating the treatment of AIS patients [9,10]. To mitigate the stroke triage process, several pre-hospital scales have been proposed to identify LVO and reduce the time metrics [11-16]. Our group has previously shown that the VES protocol is a simple and effective tool for detecting LVO in the field, by implementing it in the Ventura County of California, USA [17]. As a screening tool, VES was observed to have a sensitivity, specificity, and positive predictive value of 94.7%, 86.8%, and 58.1% respectively, with an accuracy of 84.9% [17]. This current study is a continuum where we observed a significant reduction in DGPT among patients undergoing EVT after implementation of the VES protocol as compared to patients who were triaged before VES.

Pre-hospital triage and EMS evaluation is a critical first link in the healthcare system and plays a pivotal role in the treatment of AIS patients with LVO [1,20]. Prompt and accurate evaluation facilitates optimal stroke care treatment. Our study identified that the VES protocol can swiftly diagnose LVO in the field, thereby significantly impacting the DGPT. In this study, the mean DGPT for the post-VES group was observed to be 65.5 minutes, which is remarkably shorter than the mean time of 109.6 minutes, before the implementation of the protocol. Previous studies by Zaidat et al. observed the median DGPT as 118 minutes in the TRACK registry, whereas Kasab et al., who evaluated DGPT from two tele-stroke networks observed the median DGPT of 84.5 minutes in their study cohort [21,22]. Variability in these time metrics denotes inconsistent protocols, geographical disparities and a lack of EVT-capable stroke centers, thus identifying the need for robust and accurate triage and decision-making tools to augment acute stroke treatment. 

In the US, there are nearly 37% of stroke centers with EVT capabilities which are accessible to approximately 30% of the US population within 30 minutes [23]. With the majority of the LVO patients directed to EVT incapable hospitals, stroke triage remains a critical component in stroke treatment. Patients who are transferred by other facilities for EVT, get screening and triage at the outside facility with an established diagnosis of LVO. On the other hand, patients who are brought in directly by the EMS, need to be triaged at the receiving hospital before activating LVO protocol for potential EVT. In our study we observed that with the application of the VES protocol, all AIS patients who were identified as VES positive were alerted as LVO which then triggered the activation of the interventional stroke team for potential EVT. This early activation of LVO alert from the field led to a significant decrease in DGPT at our center. This is likely due to the advance initiation of stroke protocols and coordination of neurointerventional and anesthesia teams, and the interventional suite, thereby signifying the importance of pre-arrival notification and triage protocol.

Several stroke severity scales have been developed to facilitate LVO diagnosis. Rapid Arterial Occlusion Evaluation Scale (RACE), Los Angeles Motor Scale (LAMS), Field Assessment Stroke Triage for Emergency Destination (FAST-ED), and Cincinnati Prehospital Stroke Scale (CPSS) use NIH Stroke Scale (NIHSS) elements while 3-item stroke scale (3I-SS), vision, aphasia, and neglect (VAN), and VES are more simplified screening tools to identify LVO [24,25]. While the accuracy of these scales is moderate, they are used in identifying LVO patients and supplement the triage strategy. Studies have observed that implementing pre-hospital triage may reduce the unnecessary burden of non-LVOs and augment timely intervention in suspected LVO patients [24,26]. However, further studies are warranted to validate these scales in large prospective studies.

This study reports the application and validation of an infield stroke triage tool that significantly reduces DGPT, thus implicating the necessity for a uniform protocol base approach. However, it is limited by small sample size and single-center observations. Whereas all the data were prospectively maintained, some of the study parameters were reviewed retrospectively. Moreover, our study was focused on anterior circulation LVO and subsequent iteration would benefit from including more specific symptoms related to posterior circulation. Although in our study the in-hospital stroke metrics were standardized for the VES protocol, we were not able to account for inter-hospital specific parameters. The current pilot study only included stroke codes in a single county with specific catchment areas for the EMS personnel. This may limit the generalizability to other hospitals and geographical locations and future studies will focus on a broad implementation of a coordinated stroke screening protocol.

Although this study adds to the literature, several questions remain regarding the optimal pre-hospital scale for stroke triage. This includes predictive power, accuracy, and ease of in-field implementation of the scale. Regional adoption is based on geographical location (urban/rural) or the status of the hospital (primary or comprehensive stroke center or EVT capable center), resource allocation and cost-effectiveness of a large multidisciplinary approach. 

## Conclusions

VES is a simplified screening tool for the identification of ELVO by EMS. Implementation of VES has also been shown to decrease the door-to-groin puncture time. However, future prospective multicenter studies are warranted to provide better assessment and validation at the population level. 
